# Foaming and Physico-Mechanical Properties of Geopolymer Pastes Manufactured from Post-Metallurgical Recycled Slag

**DOI:** 10.3390/ma17061449

**Published:** 2024-03-21

**Authors:** Mateusz Sitarz, Tomasz Zdeb, Katarzyna Mróz, Izabela Hager, Kinga Setlak

**Affiliations:** 1 Chair of Building Materials, Faculty of Civil Engineering, Cracow University of Technology, 31-155 Cracow, Poland; tomasz.zdeb@pk.edu.pl (T.Z.); katarzyna.mroz@pk.edu.pl (K.M.); izabela.hager@pk.edu.pl (I.H.); 2 Department of Materials Engineering, Faculty of Materials Science and Physics, Cracow University of Technology, 31-864 Cracow, Poland; kinga.setlak@pk.edu.pl

**Keywords:** foamed geopolymer, industry waste, porous materials, paste, stereology, pore size distribution, work of damage

## Abstract

This paper presents a research program aimed towards developing a method of producing lightweight, porous geopolymer composites for the construction industry based on industrial wastes. A direct method involving the addition of chemicals is currently most commonly used to produce the porous mineral structure of a geopolymer matrix. This relies on a reaction in a highly alkaline environment of the geopolymer to produce a gas (usually hydrogen or oxygen) that forms vesicles and creates a network of pores. This paper demonstrates the feasibility of producing a slag-based geopolymer paste foamed with aluminum powder, taking into account different parameters of fresh paste production: the mixing duration, its speed and the timing of foaming agent addition. The foaming process of the fresh paste in terms of the volumetric changes and temperature development of the fresh paste during the curing of the material are observed. After hardening, the physical properties (density and porosity) as well as the mechanical parameters (compressive strength and work of damage) are determined for the nine manufactured foamed pastes. Image analysis software was used to assess the porosity distribution of the material across the cross-section of the samples. The results enabled the design of the mixing procedure to be adopted during the manufacture of such composites.

## 1. Introduction

The recent literature has shown a growing interest in the study of geopolymer materials [1,2,3]. These materials appear to offer a viable replacement for OPC cement in some applications due to the reliance on industrial waste products during production [4]. It should be highlighted that geopolymer materials themselves are not inherently ‘low CO_2_’. However, by focusing on environmentally efficient mix design and careful raw material selection, significant reductions in carbon emissions can be achieved [5]. The potential use of geopolymers in civil engineering is similar to that of cementitious materials. In some cases, the use of geopolymers may even be a better choice. The possibility of using a geopolymer matrix to produce lightweight and high-temperature and fire-resistant insulation components is being explored [6,7,8]. Considering the possibility of using industrial waste to produce the material, the mechanical properties comparable to cementitious materials and the high resistance of the geopolymer matrix to fire and high temperatures, geopolymers show great potential for this application [9]. In recent years, there has been a significant research focus on geopolymer foams characterized by tailor-made pore architectures [10,11,12].

Various types of foaming agents have the potential to be used to create geopolymers with reduced density. Different foaming agent concentrations, different surfactant categories and different precursor elements affect, among other factors, the viscosity, expansion rate and surface tension. These factors contribute to variations in the resulting pore configurations [13,14]. By incorporating appropriate additives, it is possible to produce lightweight products by infusing air during the mixing process. Alternatively, another approach is to incorporate chemicals into the geopolymer mix. These chemicals react with the alkali content to produce gas, which in turn creates a foamed microstructure within the solidified material [15]. The incorporation of metals such as zinc or aluminum into the geopolymer paste results in the production of hydrogen gas. Typically, highly reactive metallic aluminum powder is used, particularly in alkaline environments [16]. This process results in the release of both an aluminate of a 1A group metal (usually Na or K) and hydrogen gas [15]. Equation (1) represents the process.

(1) $$2Al+2{Me}_{1A}OH+6H_{2}O\to 2{Me}_{1A}Al(OH{)}_{4}+3H_{2},$$

Aluminum acts as an exceptionally fast foaming agent. Compared to zinc, an equivalent molar amount of aluminum produces 50% more hydrogen gas [17]. According to Kioupis et al. [18], up to six times more zinc is required to achieve the same material density as aluminum foaming. The different foaming capacities of zinc and aluminum were also considered by Kränzlein et al. [19], who incorporated 1.0% zinc powder into the solid raw materials, while the use of aluminum was limited to a maximum of 0.2%. The literature [20,21] has documented the use of metal powders, emphasizing their properties, potential for in situ application or the relatively simple regulation of the resulting porosity levels. However, it is important to note that aluminum powder has been found to cause a delay in strength development within a geopolymer matrix. This delay is due to the formation of aluminum hydroxide gel in the early stages of the reaction, which can precipitate onto precursor particles and inhibit their dissolution [22].

Silicon powder [23,24] or alternative silicon-containing compounds such as FeSi alloys and SiC [25,26], as well as silica fume [27,28] have the ability to generate hydrogen when exposed to an alkaline environment. Within the current movement to promote environmentally conscious materials, silica fume can be viewed favorably as another waste by-product used in geopolymer synthesis. As silica fume is mainly composed of amorphous SiO_2_, the foaming mechanism is related to its impurity content. As these foaming agents, which are associated with the release of hydrogen, are associated with silicon, they often result in ultra-macroporous structures [29].

Another category of chemical agents that cause foaming are peroxides such as hydrogen peroxide [30] and organic peroxides. These substances undergo reactions that result in the release of oxygen gas [17]. Air bubbles composed of oxygen (O_2_) become confined within the paste, causing expansion and a rise in volume. Hydrogen peroxide exhibits thermodynamic instability and can readily break down into water and oxygen gas, following the reactions outlined in Equations (2) and (3) [31]:

(2) $$H_{2}O_{2}+OH^{-}\to {HO_{2}}^{-}+H_{2}O,$$

(3) $$H{O_{2}}^{-}+H_{2}O_{2}\to H_{2}O+O_{2}+{OH}^{-},$$

The production of reduced-density geopolymers using hydrogen peroxide is affected by the fine-tuning of the peroxide decomposition kinetics, resulting in the release of oxygen, and the increase in the viscosity of the geopolymer paste [32]. Unlike metal powders, hydrogen peroxide has a well-controlled decomposition and a more uniform distribution in the geopolymer paste [33,34]. This property is achieved by a gradual release of oxygen, in contrast to the rapid and powerful generation of hydrogen when aluminum is used. The rate of hydrogen peroxide decomposition can be controlled, as demonstrated by Hajimohammadi et al. [22], who found that sodium silicate allowed for a more gradual process compared to sodium hydroxide. On the other hand, Masi et al. [35] observed that aluminum powder presents the ability to create larger pores.

In addition, other chemical foaming agents are less commonly used than the ones mentioned above. The following chemicals can be mentioned as examples: sodium perborate (NaBO_3_) [36], sodium hypochlorite (NaOCl) [37], aluminum scrap recycling waste [38] and sodium lauryl ether sulfate (SLES) [39]. Although a high degree of matrix porosity can be achieved with these methods, their practical application is usually limited by complex preparation processes or higher production costs.

In summary, among the agents used to create porous structures in geopolymers, those that produce foaming gases through chemical reactions are the most commonly used. Regardless of their composition, foamed geopolymers have a high porosity, low density and low thermal conductivity. According to a review of recent studies [40], geopolymer foams can achieve bulk densities of 300 to 1500 kg/m^3^, porosities of up to 88%, thermal conductivities of 0.083–0.766 W/mK and compressive strengths in the range 0.3–56.5 MPa. It should be taken into account that the properties of particular geopolymer foam depend on the type of precursor, foaming agent, stabilizing agent and curing conditions.

The production of low-density geopolymers is challenging and it is essential to explore a refined approach for each specific foaming process. The aim is to achieve small, uniformly distributed pores while avoiding pore collapse. Foam structure formation and its durability in geopolymer pastes depends mainly on the foaming agents and surfactants.

This article presents the results of research which aimed to produce lightweight, fire-resistant components for the construction industry from metallurgical industry residues.

The main objective of the research presented is to develop a composition and process for the production of a foamed geopolymer based on post-metallurgical recycled slag. Based on the literature review, it was decided to use aluminum powder as a foaming agent. Foaming methods using metal powders are among the most widely used due to their efficiency. Among these, aluminum powder is the most commonly chosen. It is a fast-acting foaming agent. As a result, the formation of the foamed structure starts at an early stage of material setting. Particularly with fast-setting binders, it is important to form the porous structure early enough before the material hardens. Aluminum powder makes it possible to effectively reduce the density of the material. Compared to zinc powder, it can release up to 50% more gas (hydrogen) responsible for creating the porous structure of the material. This paper presents the results of testing the influence of the technological factors of geopolymer paste with the addition of aluminum powder on the density, porosity and strength of the foamed material.

## 2. Materials and Methods

### 2.1. Properties of Post-Metallurgical Recycled Slag

As a mineral precursor, post-metallurgical recycled slag was used, produced by Metallo in Belgium. Oxide composition of the slag was obtained by X-ray fluorescence (XRF) using the EDX-7200 SHIMADZU spectrometer (Kyoto, Japan). The phase composition was determined using an Aeris PANalytical X-ray diffractometer Aeris (Malvern PANalytical, Lelyweg 1, Almelo, The Netherlands). The resulting diffractograms were analyzed using HighScore Plus software (v. 4.8, Malvern PANalytical B.V., Almelo, The Netherlands). The grain size distribution of the slag particles was measured using a laser diffraction instrument, Anton Paar PSA. The “true”, i.e., skeleton density, was determined using a helium picnometer, Quantachrome Ultrapyc 1200e.

The slag was characterized by a high content of Fe_2_O_3_ (51.7%), SiO_2_ (27.6%) and Al_2_O_3_ (6.6%). Table 1 shows its oxide composition. The true density of the slag reached 3.65 g/cm^3^.

**Figure 1:**
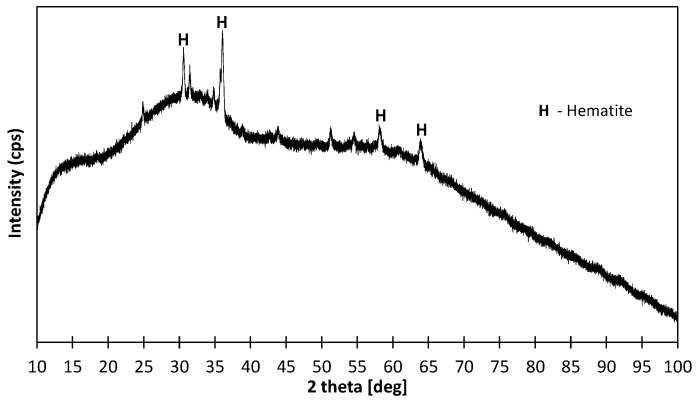
XRD diffractogram of post-metallurgical slag.

The phase composition of the slag is shown in Figure 1. Based on the XRD analysis, a mainly amorphous structure was observed. The single peaks indicated the presence of iron oxide in the form of hematite. The degree of crystallinity defined as the area under the crystalline peaks in relation to the total area was only 8.3%, which means that the amorphous solid can be estimated as over 90%.

**Figure 2:**
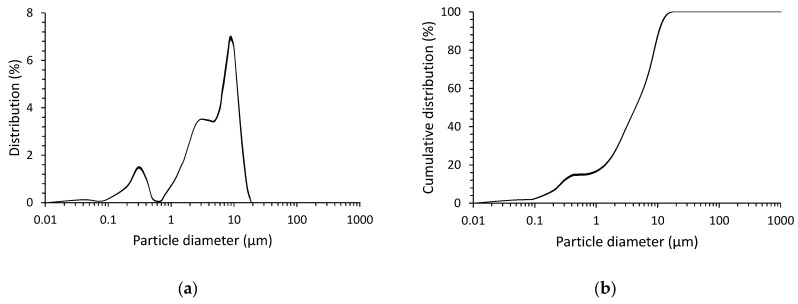
The size distribution of the slag particles: (**a**) distribution of volume shares; (**b**) cumulative distribution of volume shares.

In order to determine the particular size distribution, five measurements were made, and the results are summarized in Figure 2a,b. The diameters D_10_, D_50_ and D_90_ and the mean diameter D_mean_ were determined from the cumulative mean particle size distribution (see Table 2 and Figure 2).

The results of the particle size distribution confirm that the particle distribution is bimodal, with two characteristic peaks of 0.3 and about 9 μm. The determined grain size parameters indicate the strongly developed surface of the material. Also taking into account the degree of amorphization of the structure of this precursor, its high reactivity towards the alkaline environment of the activator can be expected.

### 2.2. Preparation of Foamed Geopolymer

The process of synthesizing a geopolymer binder involved using an alkaline solution. An aqueous solution of Woellner’s Geosil® 14517 (Woellner, Ludwigshafen, Germany) potassium silicate was used to prepare the geopolymer pastes. The solution showed a molar ratio (MR) of 1.7. This parameter is a fundamental property of aqueous sodium or potassium silicate solutions and reflects the ratio of number of moles of silica to number of moles of metal oxides. The chemical composition and key parameters of Geosil 14517 are given in Table 3, according to the manufacturer’s technical information. Extra water was added to the mixture to reduce the viscosity of the solution and improve the workability of the geopolymer paste. The optimized K_2_O and SiO_2_ content within the Geosil solution eliminated the need to introduce sodium or potassium hydroxide. This tailor-made composition met the eco-friendly requirements of geopolymer binders and streamlines the production process. The alkaline activator is the component of the geopolymer that produces the largest carbon footprint. Hydroxide manufacturing processes are highly energy-intensive. In addition, the preparation of high-concentration hydroxide solutions is inconvenient. For this reason, the consumption of alkaline substances must be optimized, preferably by using single-component, ready-to-use products.

The porous structure of the material was obtained by a chemical method. The expansion of the geopolymer mixture was achieved by adding aluminum metal powder, Al-26.98 g/mol (Chempur, Piekary Śląskie, Poland). The effect of the aluminum powder was related to the alkaline solution existing in the geopolymer paste. The aluminum reacted with the alkalines to produce hydrogen gas. The hydrogen, released as the material cured, left voids in the structure of the material. As a result, an extensive network of pores was formed within the material. Table 4 shows the composition of the foamed geopolymer per cubic meter of mix.

**Figure 3:**
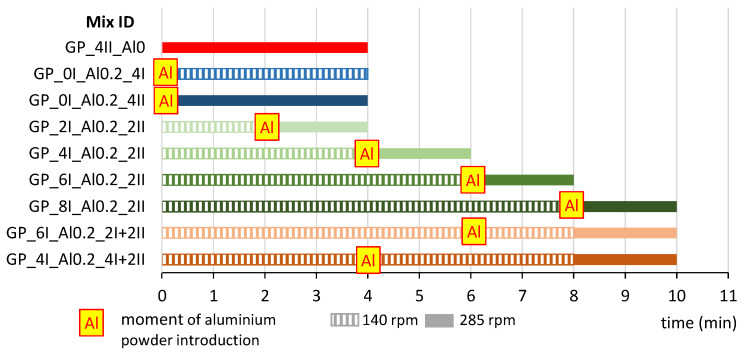
Mixing patterns of foamed geopolymer.

Due to the large number of variable parameters affecting the foamed geopolymer mixing process, some initial assumptions had to be made. Based on a literature review [41,42,43], a constant amount of aluminum powder of 0.2% by weight of the mineral precursor was approved. Different ways of mixing the paste were then analyzed. The variable parameters were (1) the mixing duration (expressed in minutes), (2) the speed of mixing (140 or 285 revolutions per minute) and (3) the time of introduction of the foaming agent. Figure 3 shows the mixing methods for all the mixes prepared. The mixing durations at each speed of mixing (axial rotation) and the time of addition of aluminum powder were presented. Nine mixes were analyzed. One reference mix was prepared without aluminum powder as a reference. The designation of the individual trial (Mix ID) is given in 4 parts separated by an underscore (_): (1) **GP** stands for geopolymer, (2) the Arabic numeral stands for the duration in minutes of mixing before the addition of aluminum powder (ex. **4**), while the Roman numeral gives the speed of mixing at this stage (**I** for 140 rpm and **II** for 285 rpm), (3) amount of aluminum powder addition (**Al0.2** in case of 0.2% by weight or **Al0** in case of not foamed mix), (4) the Arabic numeral stands for the duration in minutes of mixing after addition of aluminum powder (ex. **4**), while the Roman numeral gives the speed of mixing at this stage (**I** for 140 rpm and **II** for 285 rpm).

**Figure 4:**
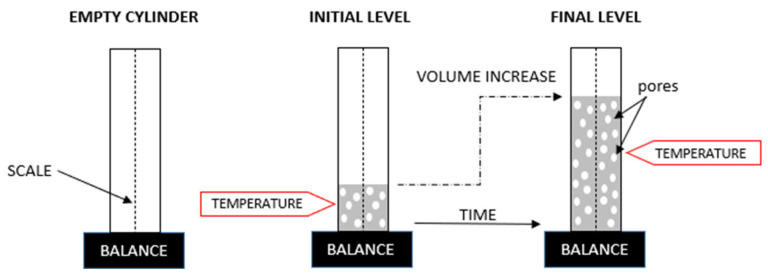
Test stand for measuring volume and temperature changes over time.

After mixing, the set volume of the fresh mixture was poured into measuring cylinders and weighed. The scale on the cylinder was used to monitor the change in volume over time. At the same time, the increase in temperature of the mixture during curing was measured at regular intervals. A schematic of the stand for measuring volume and temperature changes over time is shown in Figure 4.

### 2.3. Methods of Testing and Analysis

**Figure 5:**
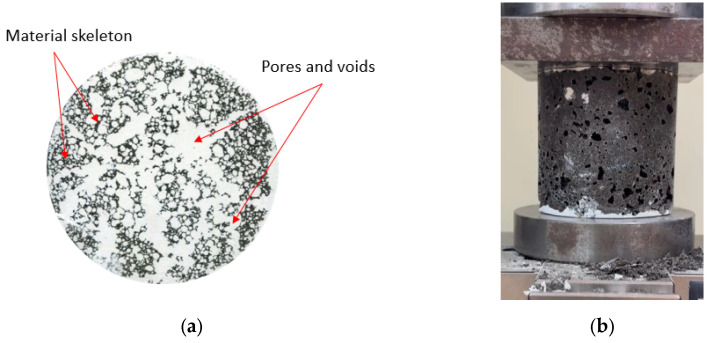
Tests of hardened foamed geopolymer: (**a**) surface preparation of specimens; (**b**) compressive strength testing.

After 24 h, the hardened samples were removed from the cylinders and stored for 28 days under laboratory conditions (20 °C, RH = 50 ± 5%). The samples were then cut into smaller cylinders equal in height to the diameter (h = ϕ = 76 mm) and weighed. In the next step, samples were prepared for porosity and compressive strength tests. The voids located on the cross-section of the specimen were filled with gypsum paste and, after a suitable time, polished. In this way, a smooth white and black surface was obtained, where the black part was the skeleton of the material and the white part was the pores (see Figure 5a). The porosity characteristics of foamed geopolymers were determined using a method based on stereological measurements. The surfaces of the samples were then scanned under constant lighting conditions at a resolution of 0.032 mm/px. The resulting images were digitally analyzed with ImageJ 1.53t software to prepare a detailed porosity characterization of the material. Image processing aims to extract useful information from digital images by manipulating them with software algorithms. In the context of the given samples, image processing aims to measure the number, perimeter and surface of pores on the cross-section. The captured image was transformed into a grey image for the convenience of thresholding, which is the process of identifying objects from the background. Several thresholding algorithms exist that are mainly based on the grey tone level histogram of the image [44]. In this particular case of foamed geopolymer composites, thanks to the use of constant lighting conditions and 20× magnification, the resulting greyscale image was transformed to binary form using a constant threshold value, which was selected manually. The threshold value on a scale from 0 to 255 was set to 165 to best reflect the course of the actual phase frontier in the composite.

Using the ImageJ software, the following porosity features were determined, where *N*_A_—the number of pixels ascribed to objects, *N_P_*—the number of pixels ascribed to their boundaries, *L_0_*—the edge length of each pixel:

- Total porosity $P_{tot}=\frac{N_{A}}{N_{tot}}\cdot 100\;[\%]$;
- The specific surface area of pores $SSA=\frac{S}{V}=\frac{4N_{P}}{\pi N_{A}L_{0}}[{\mathrm{mm}}^{-1}]$;
- Number of pores per 1 cm^2^ n [−]

Average Feret’s diameter—the longest axis that can be drawn through the object *dF* [mm]; Circularity—the number of pores expressed in % characterized by *c* > 0.9. The *c* factor equals 1 for a perfect circle and goes down as far as 0 for highly non-circular shapes $c_{i}=\frac{4\pi N_{Ai}}{N_{Pi}^{2}}[-]$.

After cross-section digitization, the samples’ compressive strengths were tested (see Figure 5b). This feature was determined on 3 specimens each time. The test was carried out using a Zwick/Roel Z50 press. This device allowed the load increment of the specimen to be adjusted by increasing the deformation over time. The test was conducted by moving the traverse at a constant rate of 1 mm/min, resulting in a constant specimen strain of 13 mε/min (10^−3^∙ε/min). Since foamed geopolymers exhibited a pseudo-plastic failure characteristic, deformation was recorded up to a value of 10 mm. Such a large deformation made it impossible to measure strain with an extensometer, so the values of deformations recorded from the traverse displacement with an accuracy of 1 µm were adopted for calculating the total damage energy *U*. Thanks to the test carried out in this way, it was possible to determine the values of the residual stresses and, as a result, the total work of damage. The value of *U* was determined from the first law of thermodynamics, assuming that no heat exchange occurred during the deformation process according to Equation (4):

(4) $$U=U_{e}+U_{d},$$

where *U_e_* means the elastic strain energy, while *U_d_* is the dissipation energy, i.e., that consumed during the internal damage and plastic deformation of the geopolymer specimen. The sum of these two energies was determined from Equation (5) and was related to the volume of the material tested:

(5) $$U=\int_{0}^{\delta_{n}}F_{i}{d\delta }_{i}=\sum_{i=0}^{i=n}\frac{1}{2}(F_{i}+F_{i+1})(\delta_{i+1}-\delta_{i}),$$

where F and δ mean force and deformation, respectively.

## 3. Results and Analysis

### 3.1. Properties of Foamed Geopolymer Pastes

#### 3.1.1. Initial Volume and Temperature Development of Fresh Paste

**Figure 6:**
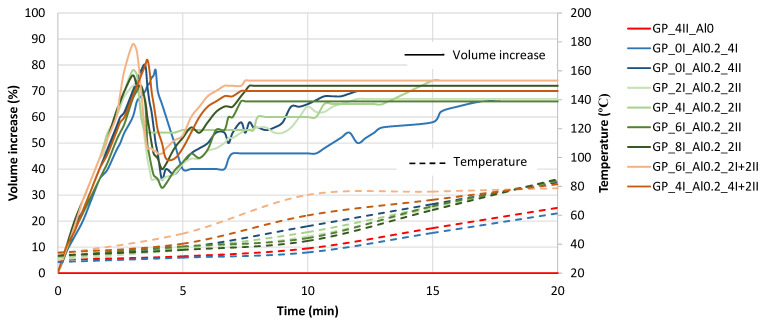
Volume and temperature changes versus the time of hardening of foamed geopolymer as a function of the mixing method.

Observations of volume changes over time and temperature measurements were made for 20 min, until the volume of the mixture in the cylinder stabilized and the material began to harden. The foaming process was entirely recorded by digital cameras. This allowed for detailed plots of the volume changes of the material over time to be generated from the video files. This relationship for each trial is shown in Figure 6. The time of 0 min in the diagram was the end of mixing and the moment of pouring the mixture into the measuring cylinders. The mechanism of change is similar for all the materials, regardless of the mixing sequence. In the first three minutes after the mixture was placed in the cylinder, the most intensive increase in volume was observed. At the third minute, there was a maximum, ranging from a 45% to about an 85% volume increase. Then, between the third and the fourth minute, a collapse of the material structure was observed, which can be seen on the graph as a decrease in the volume. In the following minutes, a recovery of the porous structure can be seen as a new increase in volume, slightly lower than the value reached at the peak before the collapse. At this stage, the materials that had a foaming agent added later in the mixing process showed a greater increase in volume. This can be observed by comparing the behavior of the composites produced with a variable initial mixing time at 140 rpm and a 2 min time at 285 rpm after dosing with aluminum powder. Although the final value of the volume increase is quite similar, regardless of the moment of the addition of the foaming agent, the composite to which the aluminum powder was added after 8 min of pre-mixing still shows the highest final value. As can be observed in Figure 6, the temperature of these mixtures with delayed Al dosage is lower during the period of volume re-growth, which favors the delayed stiffening of the composite skeleton and thus its expansion due to the formation of new pores from the released hydrogen.

The alkaline activation reaction of the tested slag was highly exothermic. This effect was enhanced by the addition of a foaming agent (aluminum powder). During the curing of the material, the mixes reached a maximum temperature in the range of 60 °C to almost 85 °C. The high temperatures caused serious shrinkage problems, resulting in the cracking of the cured material. The reference mix without the addition of aluminum powder is drawn in red in Figure 6. While comparing the temperature increase, it can be assumed that the short duration of mixing after the aluminum powder addition postpones the temperature increase and that the slow speed of the mixing results in a lower temperature being reached (in the case of mix GP_0_Al0.2_4I). Therefore, it may be stated that the increase in temperature in the first 10 min is well connected to the degree of alumina activation by the mixing process.

#### 3.1.2. Physical and Mechanical Properties of Hardened Pastes

**Figure 7:**
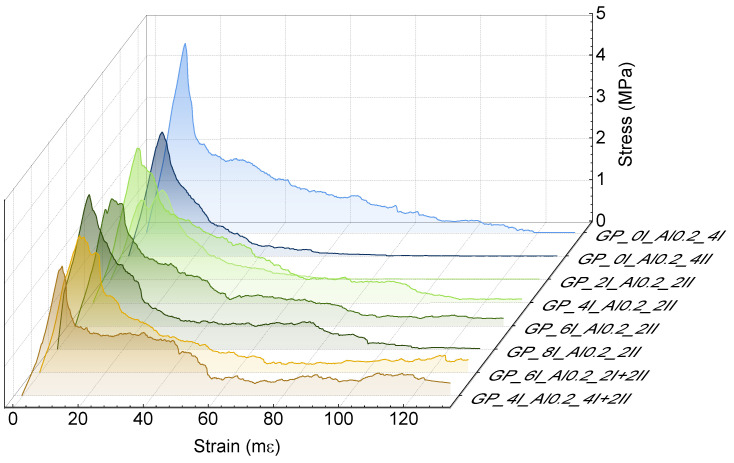
Representative stress–strain curves of foamed geopolymers produced by different mixing methods.

The failure mechanism of the foamed geopolymer pastes during the compression test is presented in Figure 7 (the reference sample in this graph is omitted due to incomparable stress values and rapid destruction). The behavior of the other geopolymers was monitored up to a fixed deformation level of 10 mm, i.e., 130 mε, because in general cases, the residual stress values in this range were positive. The graphs obtained show a pseudo-plastic failure trend in all the cases of foamed geopolymers, which allowed us to determine the work of the damage of the tested composites.

**Figure 8:**
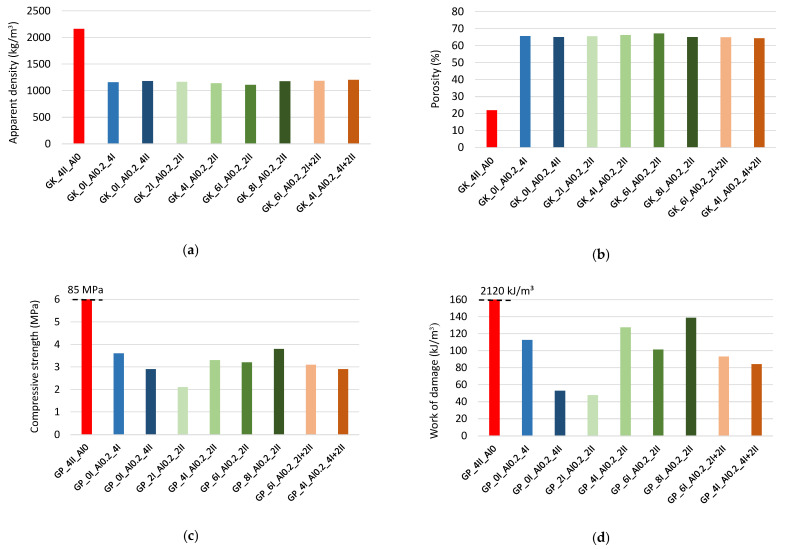
Properties of foamed geopolymers: (**a**) apparent density, (**b**) porosity—He picnometer, (**c**) compressive strength, (**d**) work of damage.

Figure 8 summarizes the properties of the GPs, presented as the average value obtained out of three samples, i.e., apparent density, total porosity (relationship between skeleton density and apparent density), compressive strength and work of damage. The apparent density was determined from the dimensions and weight of the sample after curing and drying to a constant weight at 105 °C. The reference (solid) material without a foaming agent (GP_4II_Al0) reached an average apparent density of 2400 kg/m^3^, while the foamed geopolymers were almost twice as low, around 1200 kg/m^3^. The skeleton density of the material, 3373 kg/m^3^, was measured with a helium pycnometer and used to determine the total porosity of the materials. The porosity of the non-foamed material (solid material) was about 20%, while all the foamed geopolymers had a very similar porosity of around 60%. At the same time, the foaming of the material resulted in a great reduction in the compressive strength, from a level of 85 MPa to a range between 2 MPa and 4.5 MPa. The work of the damage of the foamed material ranges from 45 kJ/m^3^ to 140 kJ/m^3^, and their values directly followed the compressive strength. The linear relationship between these characteristics, in which the value of the Pearson’s Correlation Coefficient R was 0.88 (strong positive correlation), allowed us to conclude that an increase of 1 MPa in the compressive strength entails an increase in the work of the damage of about 56 kJ/m^3^. The mechanical characteristics, i.e., the compressive strength and work of damage, were characterized by significant variability. The test results of the individual composites differed from the average value by around 20–25%.

#### 3.1.3. Detailed Analysis of Porous Structure in Hardened Geopolymer Pastes

**Figure 9:**
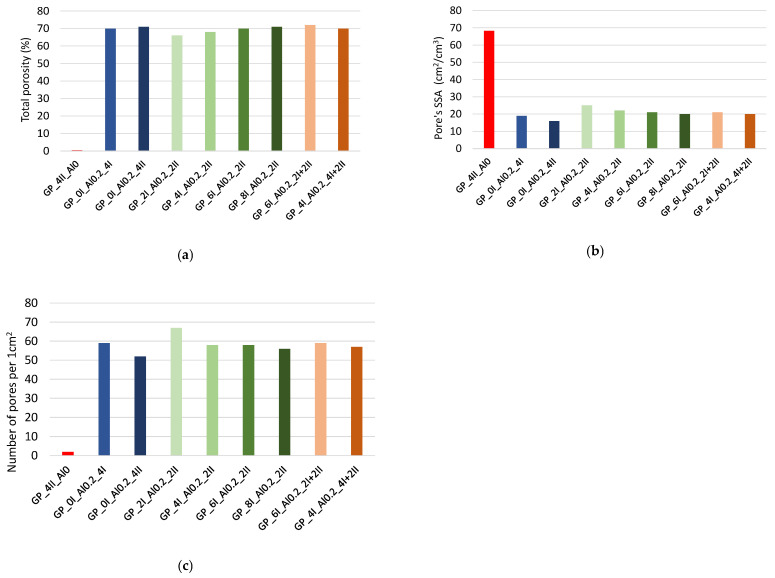
Selected parameters describing the porosity of a foamed geopolymer: (**a**) total porosity—stereological measurement; (**b**) pores’ SSA; (**c**) number of pores per 1 cm^2^.

Selected parameters of the GP’s porosity such as the total porosity, specific surface area, SSA, and number of pores per 1 cm^2^ are shown in Figure 9. The total porosity fluctuates around 70% regardless of the mixing procedure. The SSA of the pores is between 12 and 25 cm^−1^. The number of pores per 1 cm^2^ for the foamed geopolymers is in the range of 50–70. It can be seen from the presented graphs that a short mixing time of four minutes, on the one hand, allows for a high level of porosity (about 70%), but the gas released during the reaction of the aluminum with alkali accumulates in the form of large bubbles, which reduces the specific surface area of the pores to several cm^−1^, as well as the number of pores related to the unit area to about 50/cm^2^ (see Figure 9b,c). Heat conduction in such a material will undoubtedly be higher compared to that produced by mixing over a longer time and with a delayed dosage of aluminum powder. Of all the combinations tested, the maximum stereologically determined porosity (72%) was obtained with the mixing parameters of the procedure labeled GP_6I_Al0.2_2I+2II. This material had a higher specific surface area of 23.8 cm^−1^ and a pore number of about 60/cm^2^.

The variation in the Feret’s diameter obtained is small and covers narrow ranges of values from 0.45 to 0.52 mm, while the incidental technological pores in the reference material showed values of 0.28 mm. The number of circle pores characterized by c > 0.90 oscillated around the value of 60%, changing from 59 to 71%. The smallest pore diameter (Feret’s diameter) was exhibited by the material GP_4I_Al0.2_2II, while the largest was exhibited by GP_6I_Al0.2_2II. A trend was also observed for the number of circular pores increasing with a decreasing Feret’s diameter.

Using the image analysis software ImageJ, it was possible to prepare a detailed porosity characterization of the materials. Table 5 summarizes the porosity parameters determined for the selected samples, with GP_4II_Al0 as the reference and GP_6I_Al0.2_2I+2II, which was characterized by the greatest increase in volume over time. The results were based on a stereological analysis of the cross-sectional area of the samples and provide a lot of valuable information. The range of parameters that can be determined was very wide. For the reference material, an analysis of the cross-sectional area confirmed that the fraction of white pixels was only 0.4%, reflecting the porosity of the material above 32 µm. The limitation of the recorded pores was due to the resolution of the scanned surface as well as its lack of optical magnification. For this reason, the porosity value studied stereologically should not be compared with the measurement of the total porosity carried out with a helium pycnometer. In the case of the GP_6I_Al0.2_2I+2II material, the porosity determined stereologically was as high as 72%, exceeding the percolation threshold. The largest value of Feret’s diameter was 76 mm, the same as the diameter of the sample. As a later analysis of the pore size distribution showed, the maximum pore, which was a continuous phase in the composite, accounts for as much as 80% of the total porosity. The pore’s specific surface area, SSA, of the reference material was about four times larger in comparison to the foamed geopolymer. This was due to the presence of dozens of small (dF = 0.41) random pores in which the S/V ratio was 68.3 cm^−1^. In the case of the foamed material, where the cross-sectional area showed the presence of several thousand pores, the pore diameter was larger (dF = 0.50). It should be noted that the size of the pore constituting the continuous phase of the composite was not taken into account to determine the average Feret’s diameter to better characterize the size distribution of the remaining pores.

**Figure 10:**
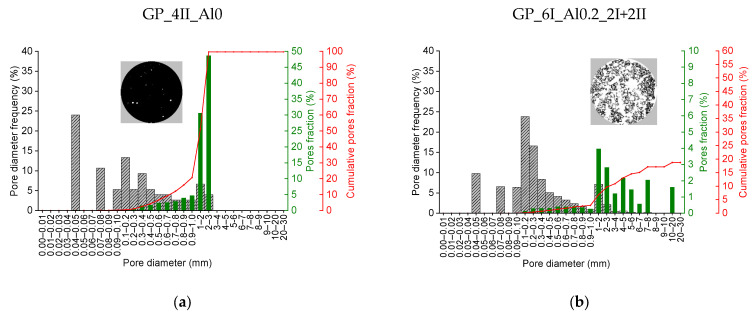
Image-based porosity analysis for (**a**) GP_4II_Al0, (**b**) GP_6I_Al0.2_2I+2II.

Figure 10 shows the distribution of the pores entrapped during the mixing of the reference material GP_0I_Al0_4II as well as the one characterized by the largest increase in volume during foaming, i.e., GP_6I_Al0.2_2I+2II. The grey color indicates the values of the pore frequency of the indicated size range. In turn, the green color indicates the contribution of individual pore fractions to the total porosity, while the red line represents the cumulative contribution of successive pore fractions. In the case of the reference material, it was possible to analyze all the pores registered, hence the cumulative curve reaches a value of 100% and in the GP_6I_Al0.2_2I+2II material, the cumulative curve ends at a value of 20%. This means that one pore with a dimension dF = 76 mm, i.e., equal to the sample diameter, accounting for 80% of the total porosity, was not included in the pore distribution analysis in order to not obscure the image of the other pores. The pore size mode in the non-foamed material, amounting to almost a quarter of all the pores, ranges from 0.04 to 0.05 mm. In the case of the foamed material, pores in the range of 0.1 to 0.2 mm are the most common, also accounting for 25%. On the other hand, the largest fraction of the total porosity in the reference material is ascribed to pores of 2–3 mm, making up 50% of it, while in the foamed material, pores of 1–2 mm make up 4% of the total porosity.

## 4. Discussion

The development of a technology to produce a foamed geopolymer material is a complex and multi-stage process. Many material and technological factors influence the final properties of the produced material. The results presented in this article relate to research aimed at developing lightweight geopolymer foams based on post-metallurgical slag. The scope of the first stage includes issues relating to the method of paste mixing (sequence of proceedings). The obtained results define the principles for further research. The use of aluminum powder as a foaming agent in the case of tested slag is an effective way of creating a porous material structure. Mixtures containing the aluminum powder achieved a total porosity of 60%. The non-foamed reference material shows 20%, which is three times less. A threefold increase in the porosity is observed with a halving of the apparent density. The binding reaction of the slag-based geopolymer with aluminum powder is very dynamic. The reaction of aluminum in an alkaline environment proceeds with the release of hydrogen. Once the reaction is initiated, the released hydrogen creates vesicles that cause the mixture to expand. The intensity of the gas released was the highest about four minutes after the end of mixing. This causes individual vesicles to cluster together, creating voids in the fresh mix. The weak skeleton of the unbound paste is unable to support the structure and the material collapses. At a later stage, it is possible to recover some of the lost volume gain. Structure collapse at an early stage of setting is a serious technological problem. Adjusting the amount of aluminum powder added may be a solution. Common amounts of aluminum powder determined from a literature review were used to prepare the mixes (0.2% by weight) [41,42,43]. The future work shall focus on the optimization of the amount of foaming agents being introduced. Another possibility is to use a chemical surfactant to stabilize the structure of the foamed material. Anionic surfactants such as sodium dodecyl benzene sulfonate and sodium oleate appear to be the suitable stabilizer for testing with the post-metallurgical slag in question [42,45]. Another challenge is the high temperature associated with the alkaline reaction of the slag activation, which is enhanced by the foaming agent. In the case of geopolymers, such elevated temperatures can create better conditions for bonding. It is known that temperatures around 60 °C–80 °C can have a beneficial effect on the formation of geopolymer synthesis products [46]. Usually, however, the analyses carried out are concerned with thermal treatments of the material over a longer period of time [47]. In this case, the aim was to verify whether the effect of self-induced heat release would be sufficient to ensure a satisfactory degree of geopolymerization of the binder and thus ensure sufficient mechanical properties of the resulting composites at the level of several MPas.

When monitoring the effect of the geopolymer paste mixing rate on the further foaming process, it was observed that applying a higher speed of mixing implies the temperature increase in the fresh mix due to the internal friction, which results in the faster activation of the aluminum powder. This causes a faster increase in the volume of the fresh paste, and a more efficient collapse and recovery effect. This is demonstrated by comparing the test results of the GP_0I_Al0.2_4I (140 rpm) and GP_0I_Al0.2_4II (285 rpm) mixtures.

By comparing the test results of GP_2I_Al0.2_2II (2 min of mixing), GP_0I_Al0.2_4II (4 min of mixing) and GP_4I_Al0.2_4I+2II (6 min of mixing), it was also noted that increasing the mixing time after the addition of Al powder usually leads to faster temperature development during the foaming process. This reduces the collapse effect and causes the recovery of volume after the collapse earlier (about 7 min). The structure of the composite stabilizes and maintains the highest level of foaming at about 70%.

However, it is important to note the negative effect of the relatively rapid temperature changes, both during geopolymerization, and especially afterwards, when the temperature of the material decreases, causing thermal stresses in the weak porous microstructure. The developed geopolymer pastes harden between 8 and 14 min after mixing. In the meantime, the paste experiences a significant volumetric change (foaming, collapsing and sometimes volume recovery again). Finally, after a short time, it becomes a rigid material, mainly due to elevated temperature. However, it is, as of yet, characterized by very low strength. The resulting thermal stresses can, therefore, easily lead to numerous microcracks in the porous structure negatively affecting further mechanical properties.

Based on the results obtained, and, in particular, the greatest increase in volume over time, a mixture of GP_6I_Al0.2_2I+2II was prepared according to the following procedure: mix the slag with the alkaline solution for 6 min at 140 rpm. After 6 min, add the aluminum powder (foaming agent) and mix for a further 2 min at 140 rpm then for two more minutes at 285 rpm. The entire mixing process takes 10 min. The resulting material has an apparent density of 1200 kg/m^3^, a total porosity of 60% and a compressive strength of 3 MPa. The properties of the material produced are similar to those of foamed geopolymers based on fly ash [18] or metakaolin [21], despite the significantly higher density of the precursor (3.65 g/cm^3^).

## 5. Conclusions

- The tested post-metallurgical recycled slag as a precursor when reacting with the activator causes the release of a large amount of energy (exothermic reaction); therefore, it is possible to induce the geopolymerization process under ambient conditions.
- The presence of aluminum powder in the geopolymer mixture causes an additional increase in its temperature, which affects the kinetics of the setting and hardening of the geopolymer composite.
- When the geopolymer paste is mixed at a higher rate, the temperature of the mixture increases as a result of the internal friction of the components, which further promotes the activation of the foaming agent additive, i.e., the aluminum powder. This is shown by comparing the test results of the GP_0I_Al0.2_4I (140 rpm) and GP_0I_Al0.2_4II (285 rpm) mixtures. As a result, the temperature of the mixture increases more quickly. No effect of the initial mixing time (140 rpm) before adding the foaming agent on the final temperature of the geopolymer mixture is observed. However, when aluminum powder is added to a mixture, increasing the mixing time usually leads to faster temperature increase during the foaming process. This was confirmed by comparing the test results of GP_2I_Al0.2_2II (2 min of mixing), GP_0I_Al0.2_4II (4 min of mixing) and GP_4I_Al0.2_4I+2II (6 min of mixing).
- Increasing the mixing time after the addition of aluminum powder and thus the greater increase in the mix temperature while foaming causes the recovery of the volume after the collapse effect to occur earlier (about 7 min), and the structure of the composite stabilizes and maintains the highest level of foaming at about 70%. In the future, it will be necessary to optimize the amount of aluminum powder added to improve the efficiency of producing a porous material structure and to reduce the adverse effects that cause the material structure to collapse in the early stages of skeleton formation. The possibility of incorporating a surfactant into the mix should be considered, as this type of additive can have a stabilizing effect on the resulting porous structure.
- The GP_6I_Al0.2_2I+2II mix showed the highest level of volume gain, at around 90%. In addition, it showed a comparable level of collapse to the other mixes, achieving the most favorable volume recovery. Therefore, this mixing procedure seems to be the most promising for further studies related to the participation of a foaming agent and the presence of stabilizers in the form of surfactants to reduce the collapse problem.
- The characterization of the pore distribution of foamed building materials, featuring a high porosity of several tens of percent and individual pore sizes expressed in µm or mm, is practically impossible with known methods for building materials, such as mercury intrusion porosimetry, MIP. Therefore, the method presented above for the stereological measurement of porosity and its distribution in relation to pore diameter appears to be a very promising method. The use of the ImageJ software and its algorithms for this process makes it possible not only to determine the total porosity or its distribution but also additional parameters such as the specific surface area and circularity of the pores.

## Figures and Tables

**Table 1:** Oxide composition of the slag.

Oxide	Content (%)	Oxide	Content (%)
Fe_2_O_3_	51.7	Cr_2_O_3_	1.4
SiO_2_	27.6	P_2_O_5_	1.2
Al_2_O_3_	6.6	MnO	0.82
ZnO	4.3	MgO	0.81
CaO	3.3	CuO	0.69

**Table 2:** Particle size distribution parameters.

**SLAG**	**Mean Particle Size (μm)**	**d_10_ (μm)**	**d_50_ (μm)**	**d_90_ (μm)**
	5.4	0.26	4.3	10.5

**Table 3:** Woellner Geosil 14517. Data provided by the manufacturer.

Characteristic	Woellner Geosil 14517	Unit
K_2_O content	21.84	wt.%
SiO_2_ content	23.5	wt.%
density	1.512	g/cm^3^
viscosity	22	mPa·s
weight ratio (WR = wt.% SiO_2_/wt.% K_2_O)	1.08	−
molar ratio (MR = mol SiO_2_/molK_2_O)	1.70	−

**Table 4:** Foamed geopolymer mix compositions.

Mix Composition	Content (kg/m^3^)
Slag	1843.0
Woellner Geosil 14517 (K—water glass)	552.9
Additional water	129.0
Al powder—0.2% by weight of slag	3.7

**Table 5:** Digital porosity analysis for foamed geopolymer paste.

GP_6I_Al0.2_2I+2II	PARAMETER	VALUE
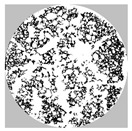	Total porosity [%]:	72.0
	Pores SSA (S/V) [cm^−1^]:	15.4
	Number of pores per 1 cm^2^:	45
	Average Feret’s diameter [mm]:	0.50
	Circularity C > 0.90 [%]:	61.9
**GP_4II_Al0**	**PARAMETER**	**VALUE**
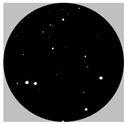	Total porosity [%]:	0.4
	Pores SSA (S/V) [cm^−1^]:	68.3
	Number of pores per 1 cm^2^:	2
	Average Feret’s diameter [mm]:	0.41
	Circularity C > 0.90 [%]:	46.7

## Data Availability

Data are contained within the article.
